# Ethical considerations for HIV remission clinical research involving participants diagnosed during acute HIV infection

**DOI:** 10.1186/s12910-021-00716-1

**Published:** 2021-12-28

**Authors:** Adam Gilbertson, Joseph D. Tucker, Karine Dubé, Maartje Dijkstra, Stuart Rennie

**Affiliations:** 1 Pacific Institute for Research and Evaluation, Chapel Hill Center, 101 Conner Drive, Suite 200, Chapel Hill, NC 27514-7038 USA; 2 UNC Center for Bioethics, Department of Social Medicine, University of North Carolina at Chapel Hill, Chapel Hill, NC 27599 USA; 3 Institute for Global Health and Infectious Diseases, University of North Carolina at Chapel Hill, Chapel Hill, NC 27599 USA; 4 Faculty of Infectious and Tropical Diseases, London School of Hygiene and Tropical Medicine, Keppel Street, London, WCE1 UK; 5 UNC Project-China, 2 Lujing Road, Guangzhou, China; 6 Gillings School of Global Public Health, University of North Carolina at Chapel Hill, Chapel Hill, NC USA; 7 Department of Infectious Diseases, Amsterdam Infection and Immunity Institute, Amsterdam University Medical Centers, Academic Medical Center, University of Amsterdam, Amsterdam, The Netherlands; 8 Department of Infectious Diseases, Public Health Service Amsterdam, Amsterdam, The Netherlands

**Keywords:** Acute HIV, Primary HIV, Early HIV, HIV remission, HIV cure, Ethics, Bioethics, Medical ethics, Clinical trials

## Abstract

HIV remission clinical researchers are increasingly seeking study participants who are diagnosed and treated during acute HIV infection—the brief period between infection and the point when the body creates detectable HIV antibodies. This earliest stage of infection is often marked by flu-like illness and may be an especially tumultuous period of confusion, guilt, anger, and uncertainty. Such experiences may present added ethical challenges for HIV research recruitment, participation, and retention. The purpose of this paper is to identify potential ethical challenges associated with involving acutely diagnosed people living with HIV in remission research and considerations for how to mitigate them. We identify three domains of potential ethical concern for clinicians, researchers, and ethics committee members to consider: 1) Recruitment and informed consent; (2) Transmission risks and partner protection; and (3) Ancillary and continuing care. We discuss each of these domains with the aim of inspiring further work to advance the ethical conduct of HIV remission research. For example, experiences of confusion and uncertainty regarding illness and diagnosis during acute HIV infection may complicate informed consent procedures in studies that seek to recruit directly after diagnosis. To address this, it may be appropriate to use staged re-consent procedures or comprehension assessment. Responsible conduct of research requires a broad understanding of acute HIV infection that encompasses its biomedical, psychological, social, and behavioral dimensions. We argue that the lived experience of acute HIV infection may introduce ethical concerns that researchers and reviewers should address during study design and ethical approval.

## Background

While exact definitions vary [1], acute HIV infection (AHI) generally refers to the brief period (circa 25 days; designated Fiebig stages 1 and 2) between initial infection and detectable HIV-specific antibodies, i.e., seroconversion [1–3]. It is during this time that individuals are most infectious [4–6], yet their HIV infections are undetectable using HIV antibody assays [7]. As a result, many people are unaware that they are living with HIV [8–10]. During early AHI, reservoirs of replication-competent viral DNA become established within the body [11–13]. These latent reservoirs comprised of memory CD4 + T [14–16] and other cells [17, 18] largely account for HIV’s current incurability, despite the effectiveness of combination antiretroviral therapies (cART) [11, 19–22]. However, initiation of cART during the earliest stages of infection may limit reservoir size [11, 23, 24]. Moreover, people diagnosed and treated with cART during AHI (referred to here as “AHI people”) tend to have better long-term prognoses than those diagnosed and treated at later stages [25]. AHI people are known to have less severe inflammatory responses [26, 27], less genetically diverse infections [28], and fewer comorbidities associated with chronic HIV infection (CHI) [29]. Additionally, research suggests that they may have faster immunological recovery [30], decreased immune activation and exhaustion and better preservation of HIV-specific immunity [31], greater potential for post-treatment HIV control, and less opportunity for viral immune escape [32, 33].

For these reasons, AHI people have long been of special interest for HIV prevention and treatment research [12, 34]. In recent years, AHI people have also increasingly become important for research aimed at achieving HIV remission [11, 19, 35, 36]. By HIV remission, we mean long-term cART-free control of viremia at undetectable levels without complete viral elimination[37], which is more feasible than viral eradication [38]. There is good reason to believe that limiting the establishment of HIV latent reservoirs in the body may be an initial, yet crucial step in the development of therapies that can consistently achieve HIV remission [39, 40]. If this proves to be the case, AHI people [41–43] may be among the first to achieve long-term, post treatment control of HIV [44].

However, AHI’s short duration, [2] combined with HIV diagnostic limitations for detecting HIV prior to antibody production, [1, 7, 45] make AHI people relatively difficult and more costly to identify and recruit for research [45–47]. Despite these challenges, several HIV remission studies involving AHI participants are in progress or completed, [48] more are planned, [49] and numerous AHI research cohorts already exist [50–57].

While these efforts have already led to a better understanding of HIV pathogenesis [35, 58], potential ethical issues associated with the inclusion of AHI people in clinical research have received comparatively less attention [59], although empirical reports are starting to emerge [60]. This gap is concerning because studies involving potentially more vulnerable participants (i.e., those more susceptible to incurring wrong via research participation) [61–63] generally require increased ethical scrutiny [64, 65]. Existing research suggests that certain social [8–10, 66–72], psychological [73, 74], and illness [23, 75] experiences associated with AHI may make some participants uniquely susceptible [76] to adverse outcomes. Yet, one question that remains is to what extent the lived experiences and needs of AHI participants vary significantly from those of people newly diagnosed or already living with CHI.

HIV remission studies involving people living with HIV are scaling up globally. The potential risks posed to participants by these studies cover a wide range, from very low (e.g., observational studies and low-risk procedures such as leukapheresis and brain MRI) to higher risk (e.g., longer-term analytical treatment interruption, experimental and/or potentially toxic interventions, invasive study procedures such as lumbar puncture, lymph node and colon biopsy, etc.). In this paper, we consider how lived experiences might differ between AHI and CHI people and identify potential ethical issues that may warrant greater scrutiny during research proposal design and review, especially for higher risk studies. Our aim is to identify points to consider for clinicians, researchers, and ethics committee members regarding the recruitment, retention, and monitoring/care of adult AHI people. Our hope is to encourage dialogue between researchers, ethicists, and other stakeholders, to inform the design and conduct of future studies, and to suggest needed avenues for further research.

## The social, behavioral, and psychological dimensions of acute HIV infection

Regardless of one’s stage of infection, receiving an HIV diagnosis is a difficult, life-changing, uncertainty-fraught experience [75]. This potentially tumultuous process may involve the formation of new HIV-positive identities [77] and negotiating what infection means to patients, their partners, and families. For those diagnosed during AHI, evidence suggests that this time is “often a marker of chaotic events in patients’ lives” [10] and, similar to HIV diagnoses generally, AHI diagnoses have been linked to distress, anxiety, depression [10], anger, confusion [78], and guilt [74]. Previous research on AHI has considered its social, emotional, and sexual hallmarks [56, 66–71, 78–80]. The National Institute of Mental Health Multisite Acute HIV Infection study [81, 82] found that AHI people reduced their numbers of sexual partners and serosorted, but continued to practice condomless sex [66]. This study also found AHI people to have a substantial burden of psychiatric morbidities, including alcohol/substance abuse disorders (though for most people, these conditions existed prior to AHI diagnosis and may therefore not be attributable to AHI [73]). The authors also noted limited awareness among people about the meaning and public health importance (i.e., increased infectiousness) of AHI [81].

Although AHI people and those diagnosed during CHI seem to have similar experiences at diagnosis, including shock, hopelessness, and detachment [83], evidence suggests that AHI post-diagnosis experiences may be somewhat different. Such differences, briefly noted below, may warrant special ethical attention during the recruitment procedures for HIV remission clinical studies.

First, during the AHI period a person has not yet begun to produce detectable levels of HIV antibodies, and it may be difficult to diagnose them accurately and definitively [7], especially in contexts lacking access to the latest diagnostic technologies [84]. During AHI up to two-thirds of people experience symptoms of infection known as acute retroviral syndrome (ARS) [10], with symptoms that include fever, rash, anorexia, body aches, fatigue, and/or headache [46, 85, 86]. These flu-like symptoms are ambiguous and easy to misattribute to other conditions [12, 46]. This, combined with the finding that many care providers are unfamiliar with AHI [8, 81], compounds known challenges in AHI diagnosis [7]. Preliminary test results interpreted as “false positive” or indeterminate may add to patient confusion [8–10] and potentially foster mistrust toward the medical system, including biomedical research. Recognition of the difficulties in diagnosing AHI has led others to call for primary and emergency care providers to have a low threshold of clinical suspicion for AHI in many settings [7, 8, 10, 82].

Second, due to the shorter duration between infection and diagnosis, AHI people may have a much better understanding of the risk behaviors or sex partners that led to their infections [8, 9, 74, 78] compared to CHI counterparts. Therefore, AHI people may have more guilt and/or clearly directed resentment toward their partners [74, 78], depending on the circumstances of the suspected route of transmission. Conversely, in the age of cART and its widely understood benefits, AHI people who perceive themselves as at higher risk for infection may feel grateful to have prompt detection of HIV infection and the opportunity to begin cART early [87].

Third, following HIV diagnoses, almost all people receive new and potentially overwhelming information concerning HIV, their health, and the healthcare system. Soon after diagnosis, individuals must make important decisions regarding their HIV care. Examples of these decisions include where to receive care, HIV status disclosure, how to protect sex/drug use partners, whether and when to begin lifelong treatment, and whether to participate in research (if offered the opportunity). In Kenya, researchers found that people’s decisions to begin cART on the same day as their diagnosis were linked to an understanding of their advantaged status as AHI people [72, 88, 89]. For those diagnosed during AHI, these decisions may be perceived as especially urgent and anxiety-ridden, given their heightened infectiousness and the long-term benefits offered by immediate uptake of cART [90, 91]. Furthermore, studies seeking to enroll AHI people often try to do so soon (if not immediately) after diagnosis. Enrollment immediately or soon after diagnosis is less often necessary within studies involving (even newly diagnosed) CHI people.

## Potential ethical issues in research involving AHI participants

Previous research has highlighted people’s experiences during AHI [8–10, 66, 68, 73, 74, 81, 92], the ethics of HIV treatment [93–95] and prevention [96–101], and the ethics of clinical research generally [102–104]. Additionally, much work has been dedicated to clinical research ethics for studies involving adults living with HIV [36, 59, 105–110], including issues related to informed consent [111–116], analytical treatment interruption (ATI) [60, 87, 109, 117–119], and risks versus benefits [120–128]. Following these efforts, we suggest three primary domains of potential ethical concern for HIV remission clinical studies involving AHI participants. These are: (1) Recruitment and informed consent; (2) Transmission risks and partner protection; and (3) Ancillary and continuing care.

### (1) Recruitment and informed consent

Depending on the intervention, trial phase, research hypothesis and other factors, some HIV remission studies currently recruit AHI participants immediately after diagnosis, while others require that AHI participants have a history of successful suppressive therapy before they are eligible to participate [49]. Given the immunological advantages of diagnosing HIV early, and as HIV remission research progresses and more promising interventions are found, it seems likely that future studies will increasingly recruit AHI people as early as possible. If this becomes common practice, one key ethical challenge might be how best to provide assistance, counseling, and support to recently diagnosed patients, who may be experiencing symptoms such as ARS, to increase their likelihood of making autonomous decisions to join or decline participation in remission studies.

Available research suggests that receiving informed consent from AHI study candidates at diagnosis may be especially challenging. For instance, studies have found that some AHI participants recalled not having understood the meaning of “acute” HIV during diagnosis, as well as difficulty absorbing information during post-diagnosis counselling [8, 72]. Describing their experiences in one study, a participant explained that, “emotionally, I wasn’t able to probably hear.” Another participant in the same study offered that, “my mind was elsewhere” when explaining why he could not remember discussing the significance of AHI [8]. Other research has shown that although participants suffering from depression can understand informed consent information, they tend to be less capable of recognizing its significance to their situations, especially concerning treatment options [129, 130].

While it remains unclear whether there should always be heightened concern about the quality of informed consent among AHI participants, researchers should at least consider the possibility that unique life experiences associated with AHI might color perceptions and/or unduly bias individuals to accept or decline research opportunities. Therefore, even when individuals have provided informed consent to participate close to diagnosis and appear to comprehend the nature of a study, this understanding may be, in some cases, suboptimal or fleeting [8, 72]. However, knowing about the mental health challenges that some AHI patients face should not lead to the assumption that mental health interventions are always needed or wanted, or that an AHI participant’s decisional capacity is necessarily compromised to an extent that justifies non-inclusion. Instead, capacity to provide valid informed consent should be assumed unless the participant (or their behavior) suggests otherwise. If researchers recognize these issues from the outset, they can plan to provide more interactive and much longer than usual sessions for informed consent [72] and/or incorporate tests of understanding and informed consent comprehension enhancement strategies [131–136]. If concerns around psychosocial issues arise, referral mechanisms for mental health services [116] should be included in a protocol's design and budget.

To ensure patients do not feel rushed to make decisions about participation, and to help mitigate the aforementioned issues related to informed consent, researchers can schedule recruitment and consent procedures for a time well after diagnosis. When this is not possible, or when doing so would undermine research quality or preclude conducting the research entirely, it may be advisable to employ staged informed consent, re-consent, or process consent procedures as alternative strategies. Staged informed consent refers to procedures involving more than one meeting, allowing participants additional time to think, discuss, and/or seek further advice prior to enrollment [137]. Re-consent means providing participants with multiple opportunities to review consent materials after enrollment and to decide whether or not to continue with participation [138]. Doing so allows participants to review at a later, calmer time their earlier decisions made at or around the time of diagnosis. As a result, participants may be able to make more informed choices concerning their continued participation in research. Process consent consists of learning more about the person before the consent process begins, assessing legal capacity, obtaining initial consent, providing opportunities for ongoing consent (e.g., frequent re-consent), and obtaining feedback and providing the participant with additional support [139]. While process consent has been championed as a more appropriate means of obtaining consent from individuals living with dementia [139], other cognitive impairments, or within the context of palliative care [140], the “person-centered” nature of this approach could offer useful insights for researchers who work with other participants whose inclusion in research may raise similar ethical concerns.

Other challenges related to informed consent may arise if AHI people’s own healthcare providers offer them opportunities to participate in research. Primary care providers are among those more likely to identify individuals in the acute phase of HIV [1, 82] and are important gatekeepers linking people to research opportunities. A familiar worry among ethicists is that people might agree to participate in research out of deference to medical authority [141], fear of losing medical benefits, or feelings of indebtedness for access to the latest experimental interventions that are unavailable to the public [142]. Unlike participants in most oncology studies, people living with HIV have access to highly effective treatment on which most can fare well for the rest of their lives [110]. As such, joining research out of deference to medical authority or fear of losing medical benefits (even if unfounded), or in order to gain access to the latest experimental interventions, could be morally worse in the case of HIV remission research involving ATI, given the effectiveness of continued cART as the standard of care. Based on concerns similar to those outlined here, the Declaration of Helsinki suggests that researchers use non-clinical personnel to recruit patients in all clinical trials [143]. To avoid conflating medical care with clinical research, individuals providing diagnoses should be different (when possible) from those offering research participation, even if some patients may prefer to be recruited by their already known and trusted care providers.

Early HIV diagnosis may inspire optimism among people about their disease progression [8, 144]. Such positive outlooks may be communicated or reinforced to people during the informed consent process [145], depending on whether and how participants are informed about why AHI is particularly important for HIV remission studies. For example, research from Thailand suggests that AHI people may come to see themselves as having bodies that are “special” to science [60, 87]. Evidence-based optimism about the long-term benefits of early-initiated cART could spill over into non-evidence based optimism (i.e., therapeutic misconception or misestimation) about the likelihood of achieving remission in early-phase trials that will not provide this benefit [105, 146, 147]. To counter this, researchers should strive to make clear to every potential participant the risks and the lack of direct medical benefit from study interventions (as appropriate), and spend more time and effort on this aim [72] than would be called for in other types of studies.

### (2) Transmission risks and partner protections

Analytical treatment interruption (ATI) is currently a necessary component within the protocols of certain HIV remission clinical studies [49, 148–150]. Analytical treatment interruption involves the temporary suspension of cART in order to assess participants’ times to viral rebound or altered viral set point, proxy measures for the efficacy of a given intervention [151]. Unfortunately, there may be multiple biological risks associated with longer duration ATI beyond any potential toxicities related to experimental interventions. These include flu-like illness (ARS), increased inflammatory immunological responses, reduced CD4 + count, reseeding of the HIV reservoir, viral drug resistance, cardiovascular, central nervous system, liver, and kidney damage, loss of the long-term immunological advantages of early cART, and decreased responsiveness to future remission interventions [109, 110, 128, 152–156]. Importantly, recent evidence suggests that short-term ATI does not result in permanent immune system damage, drug resistance, or expansion of the HIV reservoir [157]. Additionally, some participants may look forward to ATI and to “feeling normal” and taking a “break” from daily cART adherence [60].

However, longer-term ATI does introduce the risk of HIV seroconversion for participants who would have otherwise remained antibody negative due to early treatment initiation prior to seroconversion and continued cART [60]. The presence of HIV antibodies—and a positive HIV antibody test—could result in stigmatization and other social harm events. In certain countries such as Thailand, employment prospects, health insurance, and mortgage applications can be negatively affected [60]. These concerns are similar to those voiced by participants living without HIV in HIV studies involving antibody-inducing vaccines who, though they remain HIV free, may test positive via antibody-based tests [158, 159]. Participant circumstances and local contexts should therefore be considered when determining the inclusion and exclusion criteria for HIV remission study protocols involving AHI participants [151]. Such considerations have prompted suggestions to exclude AHI participants who remain antibody negative from HIV remission studies involving ATI [160].

There is no risk of sexual transmission of HIV while a person is undetectable for HIV under suppressive cART [161, 162]. However, participants who undergo ATI for research purposes (especially for extended periods) may increase the risk of HIV transmission to their partner(s) [107, 109, 110, 119, 128, 163, 164]. For participants, ATI may also introduce the potential for HIV superinfection (coinfection with a second strain of HIV) [151, 165, 166]. These transmission risks to participants and their partners should not be underestimated given the persistent high-risk social/sexual networks and evidence for continued post-diagnosis condomless sex found among some AHI people [66]. Additional social and financial risks associated with stigmatization, discrimination, and/or loss of employment may be incurred [151], as well as legal risks for participants who live within jurisdictions that have criminalized the act of knowingly transmitting or exposing others to the risk of HIV transmission [167].

From an ethics or regulatory perspective, research participants’ partners do not fit the definition of “research subjects” [168]. Despite this, researchers utilizing ATIs may have ethical and/or legal responsibilities toward third-party non-participants if doing so puts them at increased risk of HIV acquisition (if they are not already living with HIV) [169, 170]. Some ethicists have suggested that researchers should avoid conducting studies that pose serious harm to non-participants [171]. There are at least three possible positions to consider concerning the protection of participants’ partners, distinguished by the degrees of researcher intervention.

The first, and least interventionist, places the burden of responsibility to protect partners solely on research participants: researchers are under no obligation because only participants are responsible for protecting their partners. The second also places the central responsibility on research participants, while acknowledging that they may be in need of some resources and support to do so. Here, researchers could be seen as obligated to assist participants to determine their obligations to protect their partners. A third approach would shift considerable responsibility to researchers, and impose the expectation that they act directly to protect their participants’ partners, regardless of how participants’ obligations to protect are perceived. For instance, researchers could make efforts to inform their participants’ partners of potential risks, provide these partners with preventive measures, and/or obtain partners’ informed consent.

However, the latter, more interventionist approach might present additional risks such as the introduction of conflict into participants’ relationships if research personnel seek out and interact with their partners. As was learned during the vaginal microbicide trials that considered involving the male partners of female participants, policies that force researchers to inform participants’ partners about research risks are prone to deterring individuals from participating or remaining in research studies [172]. This is to say nothing of the potential complexities involved if participants have multiple partners, participants do not name some or all of their partners, partners are anonymous [173], or if a partner (or participant) does not want the relationship known to others. Would researchers need to compensate partners for the harm done to them if they acquired HIV from an ATI study participant [169]? Fortunately, a recent study suggests that participants in a trial involving ATI took seriously the increased risk of transmission and made behavioral changes to protect their partners [174].

While HIV remission researchers who utilize ATIs within their protocols should take the protection of their participants’ partners seriously, going as far as to seek them out for consent is probably too difficult and fraught, given the hypothetical situations mentioned above. That said, at the very least researchers can inform participants during consent processes about potential risks to their partners [151, 173] and provide them with condoms and/or their partners with pre-exposure prophylaxis (PrEP) [109, 110, 175] and/or referrals for regular testing, as appropriate per the context of the study and its participants [176]. Additionally, researchers who ask their participants to suspend cART should take into consideration the potential for relationship-related harms (e.g., conflict or abuse) and social, legal, and economic harms to non-participant partners such as stigmatization, privacy violations, and employment discrimination [169]. Depending on the circumstances, couples could be provided with counselling sessions to discuss and help process any risks versus benefits of participation, and/or partners could be invited to attend informational sessions to learn more about the study.

In the US, federal guidelines do not address research-related risks to non-participants [171, 173, 177, 178]. The burden to protect these non-participants (to the extent that it exists), and to consider whether to include discussions of these risks during informed consent processes, therefore falls on researchers, ethical reviewers, institutional funding bodies, and other stakeholders. Although others have previously made proposals for how the process of considering these risks and practices to mitigate them [179, 180] might be standardized [169, 173, 177], more research is needed to determine the most practical and efficient way forward.

### (3) Ancillary and continuing care

Although clinical research does not normally involve patient care in the traditional sense, HIV remission studies with AHI participants may encounter ethical challenges regarding responsibilities for ancillary care given the known associations between AHI and mental health issues [10, 73, 74], substance abuse problems [73], and other comorbidities [10, 85]. Ancillary care is care that is not required to make a study scientifically valid, to ensure a trial's safety, or to redress research injuries [181]. Ethicists (in particular, Richardson [182]) have developed theories to explain why researchers may have ancillary care responsibility towards research participants, and have identified factors determining to what extent ancillary care responsibilities are present. These include cases where participants have no alternative means to access care, care is relatively inexpensive, participants would otherwise suffer, participants and researchers have longstanding or deep personal relationships, and/or researchers owe research populations debts of gratitude. Given the relative scarcity of AHI people willing and eligible to participate in HIV remission research, and their important scientific contributions via risky studies with little potential for personal benefit, researchers may have a significant debt of gratitude toward this population.

Similarly, continuing care, or the responsibility to provide health benefits associated with study interventions after study completion, [183] is also a likely consideration in HIV remission research involving AHI participants. For instance, research interventions could eventually have relative health benefits, such as sustained viral suppression for years without treatment [58]. If AHI participants experience prolonged viral suppression following cART interruption, only to later experience viral recrudescence (and ARS), are researchers responsible to ensure participants’ access to care, even if the need for care “outlives” the study?

While this and similar questions are speculative, what remains clear is that attention and resources must be dedicated to AHI participants’ physical and mental health issues. Not only will this contribute to minimizing research risks and balancing these risks against the scientific benefits, doing so may as well contribute toward a broader ethics of care with this particular study population.

Researchers are more likely to encounter ancillary and continuing care issues in cohort-based and longitudinal studies, as well as within research that is conducted in low or middle-income countries with weaker health care infrastructures [181]. While much of this research currently takes place in wealthier countries, remission research is advancing quickly [49]. As new interventions move through trial phases, these efforts may increasingly be located in the resource-constrained countries most affected by HIV in sub-Saharan Africa and elsewhere. Thus, preparation for ancillary and continuing care may become increasingly crucial. Attention to these ethical and implementation issues ahead of proposal submissions will allow researchers to adequately plan and budget for ancillary and continuing needs for AHI participants [184]. While neither ancillary nor continuing care considerations are unique to HIV remission clinical research involving AHI people, the lessons learned by addressing questions concerning responsibilities for care will be useful to informing similar challenges in other types of clinical research.

## Conclusions

Table 1 provides an overview of the ethical themes, potential concerns, and possible ways to mitigate risks identified above. These ethical themes and potential routes of mitigation offer a primer for meaningful ethical dialogue concerning best practices for HIV remission clinical research involving AHI participants. While some of the issues raised here may be specific to AHI participants, others may apply to all HIV remission study participants living with HIV, especially recently diagnosed CHI people. However, it is important to remember one clear distinction between studies involving AHI and newly diagnosed CHI people: the urgency with which people may be recruited into research. AHI people are often asked to enroll in studies very soon after diagnosis. In HIV remission studies involving CHI people, there is not usually the same imperative to enroll immediately. Additionally, we recognize that not all remission studies carry the same risks for participants; if and how researchers and ethics committee members should respond to these potential concerns will depend on the relative balance of risks and benefits within a given study. We also acknowledge that this list is not comprehensive and further concerns may arise. For instance, the issue of whether and when to use randomized-controlled and placebo-controlled trials will become increasingly important as interventions begin showing greater signs of potential efficacy [151, 185].

**Table 1:** Ethical themes, potential concerns, and possible ways to mitigate risks in HIV remission clinical research involving potential risks for participants including analytic treatment interruption

Ethical themes	Potential concerns	Possible ways to mitigate risks
(1) Recruitment and informed consent	Attaining genuine informed consent during or soon after diagnosis	When feasible and not undermining research quality, recruitment and informed consent should not take place immediately following HIV diagnosis Offer or connect to (but do not require) immediate mental health and/or social services Provide high-quality counseling during the informed consent process (and ensure that this is included in study protocols to be reviewed by ethics committees) Staged informed consent [137], re-consent [138], or process consent procedures [139] Caution in reinforcing the importance of AHI people for HIV remission studies during recruitment and consent
	Therapeutic misconception or misestimation	Make clear statements of risks and (lack of) direct medical benefits from study interventions Include formal assessments of people’s research comprehension and implementation of strategies to improve understanding during informed consent
	Influence on participation decisions	Non-clinical personnel should approach patients about participation, not their primary caregivers, especially if the caregiver is the researcher [143]
(2) Transmission risks and partner protection for studies requiring longer duration ATI	HIV superinfection and transmission to partner(s)	Inform participants of infection risks and ask them to inform their partner(s). If possible, provide condoms and/or pre-exposure prophylaxis to participants’ partners [176]
	Unintended social, legal, and economic consequences	During informed consent discuss possible risks to partners [173] as well as the potential for harm to the participant's relationships (e.g., stigma, conflict, abuse, the end of relationships) Couples could be provided with counselling sessions and/or partners could be invited to study informational sessions Knowingly exposing others to risk of HIV transmission is criminalized in some jurisdictions. Researchers should make participants aware of legal implications as appropriate To avoid risks associated with seroconversion during ATI, researchers should consider excluding antibody negative participants from research involving analytical treatment interruption (ATI) [160]
(3) Ancillary and continuing care	Ancillary and continuing participant care	Provide care when participants have no viable alternatives, care is relatively inexpensive, participants would otherwise suffer, participants and researchers have an established relationship, and/or when the researchers owe a debt of gratitude [182]
	Mental health, substance abuse, acute retroviral syndrome, and other comorbidities	Mental health, substance abuse, and other health issues should be expected among some participants; referrals to appropriate services should be considered when indicated

As the number of HIV remission studies around the world increases, it is important to consider further the ethical concerns raised here via empirical work and ethical analyses. Significant gaps exist concerning how lived experiences during AHI might influence research participation, as well as participant wellbeing. While these gaps clearly necessitate further research, evidence suggests that research involving AHI participants, or certain subcategories thereof, may introduce distinctive ethical concerns, or amplify those already familiar to researchers and ethicists, during research design and ethical approval processes.

## Data Availability

Not applicable.

## Abbreviations

AHI: Acute HIV infection
ARS: Acute retroviral syndrome
cART: Combination antiretroviral therapy
CHI: Chronic HIV infection
